# The ultra mentality – Foundations for maximising athletic mental performance

**DOI:** 10.1016/j.jsampl.2024.100054

**Published:** 2024-02-07

**Authors:** Andrew J. Gardner, Robert Gilbert

**Affiliations:** a Discipline of Exercise and Sports Science, Sydney School of Health Sciences, Faculty of Medicine and Health, The University of Sydney, Camperdown, New South Wales, 2006 Australia; b Department of Kinesiology, Montclair State University, Normal Avenue, Montclair, New Jersey, 07043, USA

**Keywords:** Mental performance, Core beliefs, Athletic performance, Sports psychology

## Abstract

For over 100 years there has been an academic pursuit of understanding and applying the principals of sport psychology to improve athletic performance. How is it that certain athletes do their best when it means the most while others “choke”? Meeting and exceeding the challenges that are presented can completely change the trajectory of any athlete's career. What is it that separates the good from the great and the great from the superstars? Is it motivation, preparation, determination, discipline, commitment, natural talent, physical strength, or work ethic? Maybe it is a combination of all of these characteristics. Without the right mentality everything can fall apart. In Alcoholics Anonymous, it is said, “you don't have a drinking problem – you have a *thinking* problem.” The evidence is quite clear that the mental game can have a substantial impact, both for the better and for the worse, on performance. In this perspectives and viewpoints, we share our own insights from our experience for athletes to obtain the ultra-mentality and maximise their athletic performance. We have attempted to take simple words to briefly explain complicated concepts.

## 1 Introduction

**Fig. 1:**
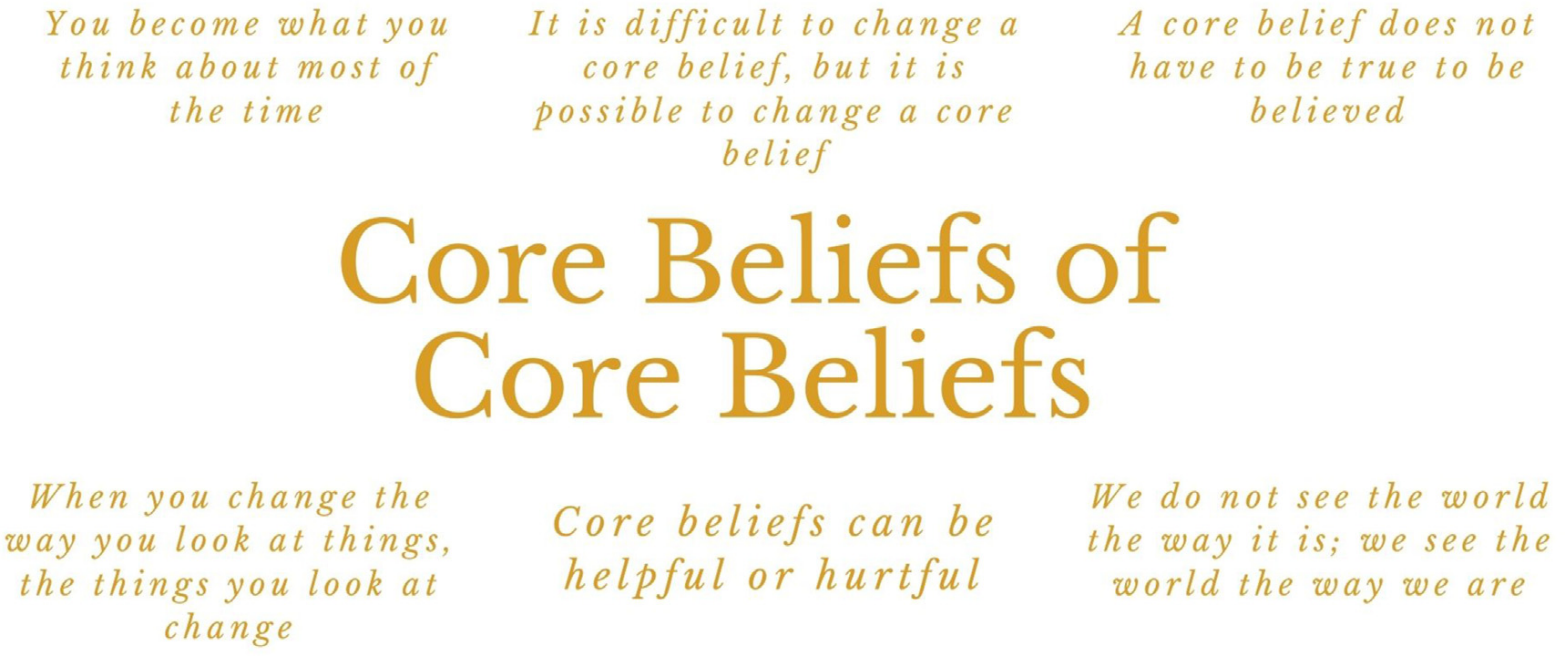
The core beliefs of core beliefs.

Sports psychology and mental performance coaching has existed in varying degrees for more than a century [[1], [2], [3], [4], [5]]. It has become common practice for elite-level athletes and teams to employ sports psychologists or mental performance coaches to give them the winning edge over their opposition [6,7], this also applies with elite player development where sports psychologists or mental performance coaches have become part of academies, and pathway programs [8,9]. Here, we provide content largely based on our own experiences (see Fig. 1).

## 2 Sports psychology and mental performance coaching

A paradox exists in sports psychology between the seemingly mutually exclusive competing interests of the physical and mental performance requirements. That is, physically an athlete in pursuit of elite performance may be required to ‘care more,’ to out-work the opponents. This perspective is captured in commonly cited quotes like: “give 110 ​%,” “There is no traffic jam on the extra mile,” and “If you do what others will not do, you will get what others will never ever get.” Conversely, from a mental perspective, the athlete executes best when they ‘care less,’ which leads to thinking less [10]. A famous line in the romantic sport comedy Bull Durham sums it up well, when the main character Crush Davis says “Don't think, it can only hurt the ball club.” Famous New York Yankees star Yogi Berra said: “A full mind is an empty bat” and Boston Red Sox Hall of Famer Carl Yastrzemski had it right when he said: “I think about baseball as soon as I wake up in the morning, I think about baseball all day. I dream about baseball all night. The only time I don't think about baseball is when I'm playing it.” How can we get athletes out of their own head? How can we get athletes to keep their minds off their minds? How can we get athletes to ‘care less’ so they can do their best when it means the most?

## 3 Creating strong core beliefs: keys to obtaining the ultra mentality

Core beliefs are our strongly engrained assumptions related to ourselves, others, and the world [11]. They are fundamental to the way we process our own reality and how we respond to situations. They are so embedded in our self-image, often nothing matters more than preserving our core beliefs. This is called belief perseverance. This can lead to challenges in an athletic environment, where confidence can fluctuate. At the elite level, behaviour in and out of competition is scrutinised, and maladaptive core beliefs can be detrimental to performance and longevity at the top. Understanding an individual's core beliefs and moving them towards positive ones is no easy task, but an essential role of a sports psychologist or mental performance coach.

## 4 Dr Gilbert's list of core beliefs of core beliefs

#1. You become what you think about most of the time.

#2. “When you change the way you look at things, the things you look at change.” – Dr. Wayne Dyer.

#3. It is difficult to change a core belief, but it is possible to change a core belief.

#4. A core belief does not have to be true to be believed.

#5. We do not see the world the way *it is*; we see the world the way *we are*.

#6. Core beliefs can be helpful or hurtful.

## 5 Core beliefs that will lead to elite performance

Here we introduce 11 key core belief statements and briefly present examples and anecdotes of the application of each of these core belief statements in real-world settings.

1. There's no failure just feedback.

World and Olympic figure skating champion Scott Hamilton estimated that it took him 40,000 falls on the ice to win his championships. This is very much like when Thomas Edison was asked how he dealt with the 14,000 failures he had before he invented the lightbulb. Edison said, “I didn't fail 14,000 times. I learned 14,000 ways of how to not invent the lightbulb!”

2. Do more than expected.

If you do more than others will do, you'll be able to get things others will never ever get.” Monica Aldama, the highly-publicised cheer coach of Navarro College, has said, “You keep going until you get it right. And then you keep going until you can't get it wrong.”

3. Because of this something good will happen

It is not what happens to you, it is how you respond to it that matters most. Dr. Terri Wurzbacher, a 74-year-old super-endurance athlete uses this mantra during her races: “Everything always works out for Terrie.” In other words, “Do not put a full stop where there should only be a comma,” or “It is not the end of the road it is just a bend in the road,” or “A setback is just a setup for a comeback.”

4. Focus on the here and now.

What place is it? What time is it? Now. Peak performance can only happen when the mind is in the present. Can you keep your mind on what you are doing while you are doing it? It is a master skill. Maybe *the* master skill. All great golfers have great focus. Tiger Woods has great focus. The “Tiger Woods” of the 1940's and 1950's was Ben Hogan. Hogan had great focus too. On the last day of a tournament, Hogan stood over a crucial putt. Suddenly, a loud train blared it's whistle in the distance. After sinking the putt, Hogan was asked if the train whistle bothered him. “What whistle?” he replied.

5. Winners lose more than losers lose.

In Major League Baseball the legendary Pete Rose set the record for the greatest number of base hits in a major league career. But before he set the record for greatest number of hits - he set the record for greatest number of outs in a career. Winners lose more than losers lose. Another example from professional baseball, Cy Young won more games than any other pitcher in baseball history. He also set the record for greatest number of losses in a career. The great Babe Ruth when asked what he thought about when he stuck out, responded “I think about hitting home runs.” He was aware that every strikeout was one at bat closer to hitting a home run. **“**Act as if it is impossible to fail” - “you are not judged by the number of times you fail but by the number of times you succeed, and the number of time you succeed is directly proportional to the number of times that you fail and keep trying.”

6. Life is a strategy game, not a talent game.

Success leaves clues, find an example of someone who has achieved all that you desire to achieve, find out how they did it, apply that strategy, and you will get what they got. All you need is already inside of you waiting for the right strategy to be applied so it can come out.

7. Self-efficacy: the believe in one's ability to successfully pursue a specific goal.

There are four fundamentally key ingredients for success and the core belief that you can achieve any specific goal that you set for yourself. You have to appreciate, understand and come to the realisation/belief that: first, you have everything inside of you that you require to succeed, you are not lacking ability, it may not be developed yet, but it is in there; second, you have all of the strategies that you need all around you, if someone else has done it, then you can do it too, you just apply the same strategy as they did; third, eliminate that voice inside your head that says “I do not feel like it.” Action is required. Fourth, analyse your results, you are successful, or you receive feedback.

8. We become what we think about most of the time.

A person's life is what he thinks about all day long. Earl Nightingale “The Strangest Secret in the World” said that a successful person is the one who is progressively realising a worthy ideal. A successful person is one who is deliberately pursuing something that they have chosen to pursue. They have set a goal, they know where they are heading, and they continue to operate in a manner that is consistent with their pursuit. The greater infatuation you have with the pursuit, the more fervently you think about it, the more likely it is that you will achieve your goal.

9. Fascinated rather than frustrated, curious rather than judgemental.

When obstacles arise, things do not turn out as planned, or the course to success does not progress the way it was anticipated to go, the importance of the response to the circumstances far exceeds the circumstances themselves. The growth mindset of having a solution-focused response, founded in fascination and curiosity, the opportunity to learn and develop, provides a platform to grow through the problem, rather than be block by it. Randy Pausch in ‘The Last Lecture’ speaks about the brick walls are there for a reason, they are not there to keep us out, they are there to show us how badly we want it, and they are there to stop people that no not want it badly enough. It is an opportunity, not an obstacle. It is an opportunity to make you better and stronger. This process over outcome focus and seeking joy in the journey builds the foundation for longevity.

10. Interested people watch obsessed people change the world.

The late Kobe Bryant described his approach in the Mamba Mentality where he highlighted five attributional pillars for success: fearlessness, relentlessness, passion, obsessiveness, and resilience. He was not interested in winning championships; he was obsessed by it. He trained so hard that the game became easy. He was so particular in his preparation that he was warming up for a game once, and something was not right. He asked the courtside staff to check the height of the ring, they said no it was right, but he insisted that they check it. It was off by one-eighth of an inch. Being so meticulous in his practice and preparation, obsessed with the process, he was able to detect the most minuscule difference that would have otherwise gone unnoticed. Obsession leaves no margin of error it covers off on every little detail. Athletes that are obsessed train and prepare harder than their competition, so that they become unstoppable. They know that they have done everything that they can to prepare to execute at the highest level they are capable of when it is required.

11. Your thoughts determine what you want, your actions determine what you get.

To this point we have emphasised the importance of what you think about. Many people who spend time thinking about their goals have established plans to achieve them (process goals), but nothing happens in the absence of action. K – A ​= ​O. Knowledge minus action equals nothing. Plan your work, then work your plan. This includes controlling the little voice inside of you that asks: ‘what will other people say?’ and ‘what will other people think?’ You need to talk to that little voice more than you listen to it. Fill it with affirmations and core beliefs that build, and do not tear down, performance.

Athletes who possess strong positive core beliefs and a growth mindset, are more resilient and equipped to navigate the long and often tumultuous journey of striving to reach the elite level or to transform themselves into a master in their chosen skill set. Establishing a healthy belief perseverance enables an athlete to remained focused on the process of continuing to grow their abilities which simultaneous protects their self-image and reduces the inner turmoil that may emerge when core beliefs are challenged or when an athlete's behaviour or thoughts do not align with their core beliefs. The ability to bring awareness to the athlete, and also to identify and to work towards positive core beliefs where they are lacking, is one of the fundamental roles of a sports psychologist or mental performance coach. While the field of mental performance has grown and become increasingly popular, the mental game remains a resource that is underutilised in many athletic environments.

In summary, core beliefs are our strongly engrained assumptions about ourselves, others, and the world. In the athletic setting, it is critically important to possess positive core beliefs. It is possible to change/modify our core beliefs. In this ‘perspectives and viewpoints’ article we have provided some of the most valuable core beliefs from our years of experience working with athletes of all levels.

## Ethics

No ethical considerations for this “perspectives” article.

## Funding

There is no funding to declare for this project.

## Declaration of competing interest

Andrew J Gardner has a clinical practice in neuropsychology involving individuals who have sustained sport-related concussion (including current and former athletes). He has been a contracted concussion consultant to Rugby Australia. He is a member of the World Rugby Concussion Working Group, and a member of the Australian Football League Concussion Scientific Advisory Committee. He has received travel funding or been reimbursed by professional sporting bodies, and commercial organisations for discussing or presenting sport-related concussion research at meetings, scientific conferences, workshops, and symposiums. He has previously been a scientific advisor for HitIQ, Ltd. Previous grant funding includes the NSW Sporting Injuries Committee, the Brain Foundation (Australia), an Australian-American Fulbright Commission Postdoctoral Award, a Hunter New England Local Health District, Research, Innovation and Partnerships Health Research & Translation Centre and Clinical Research Fellowship Scheme, and the Hunter Medical Research Institute (HMRI), supported by Jennie Thomas, and the HMRI, supported by Anne Greaves. AJG is supported by a National Health and Medical Research Council (NHMRC) Investigator Grant. He acknowledges unrestricted philanthropic support from the Tooth Foundation and the National Rugby League for research in retired professional rugby league players.

Robert Gilbert has nothing to disclose.
